# Simvastatin-Mediated Nrf2 Activation Induces Fetal Hemoglobin and Antioxidant Enzyme Expression to Ameliorate the Phenotype of Sickle Cell Disease

**DOI:** 10.3390/antiox13030337

**Published:** 2024-03-11

**Authors:** Caixia Xi, Chithra Palani, Mayuko Takezaki, Huidong Shi, Anatolij Horuzsko, Betty S. Pace, Xingguo Zhu

**Affiliations:** 1 Department of Pediatrics, Division of Hematology/Oncology, Augusta University, Augusta, GA 30912, USA; cxi@augusta.edu (C.X.); cpalani@augusta.edu (C.P.);; 2 Georgia Cancer Center, Augusta University, Augusta, GA 30912, USAahoruzsko@augusta.edu (A.H.)

**Keywords:** sickle cell disease, simvastatin, Nrf2, enhancer of zeste homolog 2, histone methylation, fetal hemoglobin, oxidative stress

## Abstract

Sickle cell disease (SCD) is a pathophysiological condition of chronic hemolysis, oxidative stress, and elevated inflammation. The transcription factor Nrf2 is a master regulator of oxidative stress. Here, we report that the FDA-approved oral agent simvastatin, an inhibitor of hydroxymethyl-glutaryl coenzyme A reductase, significantly activates the expression of Nrf2 and antioxidant enzymes. Simvastatin also induces fetal hemoglobin expression in SCD patient primary erythroid progenitors and a transgenic mouse model. Simvastatin alleviates SCD symptoms by decreasing hemoglobin S sickling, oxidative stress, and inflammatory stress in erythroblasts. Particularly, simvastatin increases cellular levels of cystine, the precursor for the biosynthesis of the antioxidant reduced glutathione, and decreases the iron content in SCD mouse spleen and liver tissues. Mechanistic studies suggest that simvastatin suppresses the expression of the critical histone methyltransferase enhancer of zeste homolog 2 to reduce both global and gene-specific histone H3 lysine 27 trimethylation. These chromatin structural changes promote the assembly of transcription complexes to fetal γ-globin and antioxidant gene regulatory regions in an antioxidant response element-dependent manner. In summary, our findings suggest that simvastatin activates fetal hemoglobin and antioxidant protein expression, modulates iron and cystine/reduced glutathione levels to improve the phenotype of SCD, and represents a therapeutic strategy for further development.

## 1. Introduction

Sickle cell disease (SCD) is an inherited hematological disorder caused by an A to T mutation in the adult β-globin gene leading to the production of adult hemoglobin S (HbS) which polymerizes under hypoxic conditions [1,2]. The polymerization of HbS is the primary event in the pathogenesis of SCD leading to chronic hemolysis, oxidative stress which contribute to stress erythropoiesis [3,4,5,6,7], and systemic hypoxia [8,9,10,11]. Although progress has been made recently, treatment options for SCD remain limited. Two critical strategies to treat SCD include γ-globin gene reactivation and reversal of oxidative stress [12]. For decades, hydroxyurea was the only FDA-approved treatment option for SCD [13] until the recent approval of Endari (oral L-glutamine) [14], crizanlizumab [15], and voxelotor [16]. Long-term experience with hydroxyurea has shown improvement in the clinical course, primarily through fetal hemoglobin (HbF) induction, but about 30% of SCD patients are not responsive. By contrast, Endari reverses oxidative stress by increasing the NADH and NAD redox potential and decreasing the endothelial adhesion of sickle red blood cells [17]. However, there are multiple sources of pro-oxidant events that contribute to the clinical phenotype of chronic systemic oxidative stress in SCD such as increased generation of free radicals [18,19], elevated mitochondrial respiratory chain activity [20,21], and blood cell auto-oxidation [22]. Therefore, additional therapeutic agents are under development for the treatment of SCD [23], mainly targeting HbF induction, anti-red blood cell adhesion, pain alleviation, oxidative stress, or inflammation. Unfortunately, the complex pathophysiology of SCD makes it unlikely that a single agent will prevent all SCD complications [24,25].

The transcription factor Nrf2 (Nuclear factor (erythroid-derived 2)-like 2) is the master regulator of the cellular oxidative stress response, which regulates a variety of antioxidant genes [26]. Nrf2 expression is regulated at the protein level through three distinct signaling pathways comprising KEAP1 (Kelch-like ECH-associated protein 1), Hrd1, and β-TrCP (β-transducin repeat-containing protein), which promote Nrf2 proteasomal degradation by different mechanisms. KEAP1 is the main regulator of Nrf2 degradation through direct protein interactions. Under resting conditions, Nrf2 is sequestered in the cytoplasm by KEAP1 and directed for ubiquitination by the E3 ligase complex. Multiple small chemical compounds such as tert-Butylhydroquinone (tBHQ), diethylmaleate, and 2-cyano-3,12-dioxo-oleana-1,9(11)-dien-28-oic acid (CDDO-Im) modify KEAP1 and disrupt its interaction with Nrf2, leading to Nrf2 nuclear translocation and antioxidant gene activation [27]. In addition, Hrd1- and β-TrCP mediate Nrf2 degradation, and pharmacologic inhibition of their expression by all-trans retinoic acid [28], LS-102 [29], SB216763 [30], and 2,4-dihydropyrano [2,3-c]pyrazoles [31] promotes Nrf2 protein stability and subsequent activation of the antioxidation response. Of these three pathways, the KEAP1-Nrf2 interaction has been most extensively investigated for its role in erythropoiesis, iron hemostasis, and heme metabolism through the expression of AMBP, ABCB6, FECH, HRG1, FTL, FTH1 [32], and two critical regulators of globin gene expression, BCL11A and KLF1 [32,33].

Notably, genetic evidence has shown that Nrf2 activation can specifically mitigate the severity of hemolytic anemia and systemic and local inflammation in transgenic SCD mice via suppressing the pro-inflammatory response and reducing reactive oxygen species (ROS) stress [34,35]. We demonstrated that Nrf2 ablation exacerbates the symptoms of SCD by decreasing the expression of antioxidant factors and the globin genes [36,37]. Indeed, Nrf2 was found to mediate γ-globin gene transcription in human normal and sickle erythroid progenitors [38] through binding the proximal promoter antioxidant response element (ARE) and the locus control region’s hypersensitive sites to facilitate a chromatin looping structure during erythropoiesis [39]. We further investigated the significant role of Nrf2 in mediating the ferroptosis stress response in SCD erythroblasts and transgenic animal models. Nrf2 ablation induced iron overload in SCD and metabolic reprogramming to induce histone hypermethylation in erythroblasts and suppressed antioxidant genes to increase lipid oxidation [37]. These findings indicated that the Nrf2 signaling pathway contributes to the regulation of the globin genes and the antioxidant stress response during erythropoiesis.

To explore HbF induction and enhanced antioxidant capacity for SCD treatment through the Nrf2 signaling pathway, our group and others have conducted investigations to discover agents that mediated Nrf2 activation. However, the clinical trial of the Nrf2 activator Bardoxolone methyl (CDDO-Me) in patients with chronic kidney disease showed the development of adverse cardiovascular events [40,41]. Hitherto, the efficacy–toxicity profile generated by different Nrf2-activating drugs has unique pharmacokinetic, pharmacodynamic, toxicokinetic, and toxicodynamic profiles. Therefore, repurposing FDA-approved drugs as novel SCD treatment options has been our focus. Previously, dimethyl fumarate (DMF), the methyl ester of fumaric acid that disrupts the interaction between Keap1 and Nrf2, was FDA-approved for the treatment of multiple sclerosis. We later demonstrated the ability of DMF to mediate Nrf2 release and nuclear translocation to activate γ-globin and antioxidative genes [27].

Other agents such as simvastatin and t-butylhydroquinone can induce γ-globin gene transcription through Nrf2 activation in tissue culture systems [33,42]. Moreover, simvastatin is an inhibitor of hydroxymethyl-glutaryl coenzyme A reductase, which activates the PI3K/Akt pathway to suppress GSK-3 activity and Nrf2 stabilization [43,44]. In addition to activating the Nrf2 signaling pathway, simvastatin mediates chromatin modifications via inhibition of histone deacetylase and DNA methyltransferase [45]. Simvastatin was also found to reduce chromatin accessibility at transcriptional enhanced associate domain elements to repress endothelial-to-mesenchymal transition to confer protection against endothelial dysfunction [46]. Moreover, simvastatin was demonstrated to inhibit HDAC1/2 and induce histone hyperacetylation to promote p21 expression and regulate tumor growth [47,48]. Notably, the efficacy of statins in the treatment of type 2 diabetes was attributed partially to DNA hypomethylation at the DHCR24, FAM50B, SC4MOL, AHRR, and ABCG1 gene loci [49,50]. Statins can also reverse subtelomeric methylation status in age-related diseases such as diabetes and inhibit Smad 6 and Smad 7 induction in autoimmune diseases [51,52]. However, whether simvastatin facilitates a disease-modifying effect in SCD and the molecular mechanism involved has not been studied.

In this study, we aim to demonstrate the effect of simvastatin to mitigate HbS sickling and ROS stress levels in SCD erythroblasts in vitro and in SCD transgenic mice. Our mechanistic studies demonstrate the ability of simvastatin to upregulate Nrf2 signaling and silence enhancer of zeste homolog 2 (EZH2)-regulated histone methylation in mediating chromatin structure modifications to activate γ-globin and antioxidant gene transcription. Our findings support the development of the FDA-approved drug, simvastatin, as a therapeutical option for treating SCD.

## 2. Materials and Methods

### 2.1. Chemicals and Reagents

Hydroxyurea (HU), dimethyl fumarate (DMF), dimethyl sulfoxide (DMSO), and H_2_O_2_ were obtained (Sigma-Aldrich, St. Louis, MO, USA). Simvastatin (SIM) was purchased from Thermo Fisher Scientific (Hampton, NH, USA).

### 2.2. Culture of Human SCD Patient Erythroblasts and H_2_O_2_ Exposure

Erythroid progenitors were generated from CD34+ stem cells isolated using microbeads selection of SCD patients’ peripheral blood mononuclear cells [44]. The anonymous collection of discarded blood samples from SCD patients undergoing chronic transfusion was classified as exempt by the Institutional Review Board at Augusta University and did not require informed consent. SCD patients’ CD34+ cells were cultured in a modified Fibach two-phase liquid culture system to generate erythroblasts at distinct stages of maturation [53]. Briefly, during phase 1, cells were grown in Iscove’s Modified Dulbecco medium with 15% fetal bovine serum, 15% human AB serum, 10 ng/mL interleukin-3, 50 ng/mL stem cell factor, and 2 IU/mL erythropoietin (EPO; Sigma-Aldrich, St. Louis, MO, USA). Phase 2 was initiated on day 7 with a similar medium without stem cell factor and interleukin 3. Chemical treatment of SCD erythroblasts was conducted on day 10 of culture and analyzed on day 12. Treatment with H_2_O_2_ at 40 µM for 8 h was completed where indicated, along with the different chemical treatments.

### 2.3. Animal Treatment

The preclinical Townes SCD mouse model (B6;129-Hba^tm1(HBA)Tow^Hbb^tm2(HBG1,HBB*)Tow^/*Hbb^tm3(HBG1,HBB)Tow^*/J) was characterized previously [54]. The experiments using the SCD mouse model were performed after approval by the Augusta University Institutional Animal Care and Use Committee. Two-to-three-month-old SCD mice (both sexes) were treated by intraperitoneal injections of HU (50 mg/kg), DMF (50 mg/kg), SIM (7.5 mg/kg), or water daily, 5 days a week for 4 weeks. Peripheral blood was drawn at weeks 0, 2, and 4. At the end of the treatment, the mice were euthanized, and spleen and liver were harvested, snap-frozen, and stored for iron, NADPH, NADP+, GSSG, and GSH levels or fixed with 1% formaldehyde for tissue pathology analysis.

### 2.4. Complete Blood Count and Differentials

Peripheral blood was collected in BD Vacutainer EDTA tubes through tail bleeding and automated complete blood count and differentials were completed on the Micros 60 CS/CT machine (HORIBA Medical/ABX Diagnostics, Irvine, CA, USA) according to the manufacturer’s protocol.

### 2.5. Isolation of Mouse Spleen CD71+ Erythroblasts and Reticulocytes

Single-cell suspensions of SCD mouse spleen were prepared and CD71+ erythroblasts and reticulocytes were isolated after labeling with biotinylated CD71 antibody followed by streptavidin MicroBeads separation with MS Columns (Miltenyi Biotec, Auburn, CA, USA) as we previously reported [37]. The single cells were processed for RNA, protein, and chromatin analysis.

### 2.6. Cell Proliferation and Viability

SCD erythroblasts at day 10 of culture were seeded at a density of 5 × 10^5^ cells/mL in Phase 2 complete culture medium and treated with indicated concentrations of chemicals. On day 12 of the culture, cells were collected and evaluated for viability by trypan blue staining.

### 2.7. Flow Cytometry Analysis

Flow cytometry was performed to measure ROS using 2′-7′-dichlorodihydrofluorescein diacetate (DCFH-DA) (Invitrogen, Waltham, MA, USA) staining. Cells were incubated with 10 μM DCFH-DA in complete culture medium for 30 min, washed twice with phosphate-buffered saline, and ROS levels were measured by flow cytometry analysis.

To measure the percentage of HbF-expressing cells (%F-cells), erythroblasts were fixed with 1% formaldehyde, permeabilized with 0.4% Triton X100, and stained with FITC anti-HbF antibody (Supplementary Table S1); isotype control IgG was used to detect non-specific staining. To measure the percentage of %F-cells in mouse peripheral blood, samples were fixed with 0.1% glutaraldehyde and permeabilized before staining with isotype control or FITC-HbF antibodies similarly.

### 2.8. RBC Sickling Analysis

In vitro RBC sickling analysis was performed as previously published [38]. Briefly, day 12 SCD erythroblasts with different chemical treatments were cultured under hypoxic conditions (1% O_2_, 5% CO_2_) for 14 h and fixed with 4% formaldehyde before moving to normal air. Using light microscopy, the number of sickle red blood cells was quantified by changes in cell morphology. Bright-field images at 20× magnification were obtained on an EVOS cell imaging system (Thermo Fisher Scientifics, Hampton, NH, USA). Areas were randomly selected where a single layer of erythrocytes was observed, and 500 cells/per treatment condition were counted in triplicate and the percentage of sickle cells was calculated based on total red blood cells counted [55].

### 2.9. Quantitative RT-PCR

Total RNA was extracted from cells using Trizol (Invitrogen, Waltham, MA, USA), reverse-transcribed, and analyzed by quantitative PCR (qPCR) using gene-specific primers (Supplementary Table S2) with SYBR Green Supermix (Biorad). Relative levels of gene expression for both target and reference genes were calculated by the 2^−ΔΔCt^ method based on Ct values. Data were presented as the mean ± standard deviation (SD) of fold change in expression. All mRNA levels were normalized to endogenous β-actin expression.

### 2.10. Western Blot Analysis

The whole-cell extracts from cultured SCD erythroblasts or isolated SCD mouse spleen CD71+ cells were prepared in RIPA buffer with protease inhibitors (Sigma-Aldrich, St. Louis, MO, USA). For histone extract preparation, the cells were homogenized in Triton extraction buffer (PBS containing 0.5% Triton X-100 and 2 mM phenylmethylsulfonyl fluoride) for nuclei isolation, and nuclear proteins were prepared by 0.2 N HCl acid extraction followed by neutralization with 1/10 volume of 2 M NaOH. Protein concentrations of the whole-cell and histone extracts were determined using the Bradford assay.

Whole-cell protein extract (30 μg) and histone extract (1 μg) were denatured, subjected to SDS-PAGE, and transferred to nitrocellulose membranes. After blocking with 5% nonfat dry milk in Tris-buffered saline and 0.1% Tween (TBS-T), the membranes were incubated with the indicated antibodies overnight at 4 °C. β-actin and histone H3 were used as loading controls. After 1 h incubation with horseradish peroxidase-conjugated anti-Mouse or anti-Rabbit secondary antibodies (Santa Cruz Biotechnology, Dallas, TX, USA), the membrane was washed and visualized by chemiluminescence in a FUJIFILM LAS-3000 system or GE Amersham ImageQuant 800 and quantified with ImageJ software (version 1.54h).

### 2.11. GSH and GSSG Assay

The levels of reduced (GSH) and oxidized (GSSG) glutathione were measured using a GSH/GSSG-Glo™ Assay Kit (Promega, Madison, WI, USA). Briefly, cultured SCD erythroblasts were washed with PBS once followed by three freeze–thaw cycles in ice-cold PBS containing 2 mM EDTA. The extracts were centrifuged, and the supernatant was collected and used immediately for the assay according to the manufacturer’s instructions. Luminescence was read by a Victor3 Multilabel Counter (Perkin Elmer, Waltham, MA, USA) and GSH and GSSG levels were calculated after normalization to cell numbers.

### 2.12. NQO1 Activity Assay

The cultured SCD erythroblasts were washed twice with PBS and assessed for NQO1 activity in whole-cell extracts following reduction of menadione with cofactor NADH and WST1. The samples were assayed at 440 nm absorbance using a NQO1 Activity Assay Kit (Abcam, Waltham, MA, USA), according to the manufacturer’s instructions. The NQO1 activity was normalized to protein content.

### 2.13. Measurement of Intracellular Cysteine Levels

Day 12 cultured erythroblasts were harvested, washed with PBS buffer once, and resuspended in distilled water. The intracellular amino acids were extracted by boiling for 10 min. The supernatant was collected after centrifugation and protein content was determined. The levels of cysteine in the supernatant were measured with a Cysteine Assay Kit (Sigma-Aldrich, St. Louis, MO, USA) along with the cysteine standards. Cysteine levels after different treatments were compared after normalization to cell numbers.

### 2.14. Cystine Uptake Assay

The cystine uptake assay was conducted with BioTracker Cystine-FITC Live Cell Dye (Sigma, St. Louis, MO, USA). Briefly, SCD day 10 erythroblasts were cultured in a complete IMDM medium with EPO (2 IU/mL) and treated with DMSO or simvastatin for 48 h. Cystine-FITC was added to cultured SCD erythroblasts at 200 µM for 45 min, allowing uptake, then the cells were collected and washed followed by quantification of cellular levels of Cystine-FITC by flow cytometry analysis.

### 2.15. EZH2 Gene Knockdown Analysis

On day 2 of culture, SCD progenitors were transduced with lentiviral EZH2 shRNA particles and selected with puromycin until analysis. Two different shRNA-targeting sequences including ugacuucugugagcucauugc and guuuguuggcggaagcgugua (Supplementary Table S2) were constructed in pLKO.1 (Addgene, Watertown, MA, USA). A standard scramble sequence (shControl) targeting a non-specific sequence ccuaagguuaagucgcccucg was used as a control. The sh*EZH2* knockdown efficacy was determined by Western blot analyses.

### 2.16. Chromatin Immunoprecipitation (ChIP)

ChIP assays were performed with indicated antibodies (Supplementary Table S1) using gene-specific primers (Supplementary Table S2) as previously reported with minor modifications [39,56]. Samples were immunoprecipitated with anti-NRF2, anti-TATA-binding protein (TBP), anti-RNA polymerase II (Pol II), and H3K27Me3 antibodies (Supplementary Table S1). ChIP DNA was quantified by qPCR with gene-locus-specific primers to determine the pull-down signals. Rabbit normal IgG was used as a non-specific antibody control.

### 2.17. NADP+/NADPH Levels

Mouse spleen CD71+ cells (2 × 10^6^) were washed with ice-cold PBS and homogenized in NADP or NADPH extraction buffer before being assayed with EnzyChrom™ NADP/NADPH Assay Kit (BioAssay Systems, Hayward, CA, USA) according to the manufacturer’s instructions. The extracts were heated at 60 °C for 5 min and then 20 μL of assay buffer and 100 μL of opposite extraction buffer were added to neutralize the extracts. The levels of NADP+/NADPH in the supernatant were determined by adding enzymes provided in the kit. Absorbance at 570 nm was measured according to the manufacturer’s instructions using a Synergy H1 hybrid plate reader (Biotek, Winooski, VT, USA) to calculate NADP+/NADPH concentrations.

### 2.18. Histology, Immunohistochemistry, and Image Analysis

Mouse spleen and liver tissues were fixed in 10% neutral-buffered formalin, dehydrated, and embedded in paraffin. Tissue sections (5 μm thickness) were subjected to hematoxylin and eosin (H&E) staining and subsequent Prussian blue staining (IHCworld, Ellicott City, MD, USA) to visualize iron deposition.

### 2.19. Iron Content

Approximately 20 mg of mouse spleen and liver tissues was homogenized in 1 mL of distilled water and supernatants were collected by centrifugation at 4 °C. The iron content in the supernatant was measured by an Iron assay kit (Bioassay Systems, Hayward, CA, USA) and iron levels were normalized to protein content.

### 2.20. Statistical Analysis

Data from at least three independent biological replicates were reported as the mean ± SD. Statistical differences were determined by an unpaired Student’s *t*-test or by two-way ANOVA with the corresponding two-tailed significance (*p* value) determined. Statistical analysis was performed using GraphPad Prism 9 software (GraphPad Software Inc., San Diego, CA, USA) and differences were considered significant at *p* < 0.05.

See the Supplementary Materials for additional methods.

## 3. Results

### 3.1. Simvastatin Activates γ-Globin Gene Expression and Reverses Sickling of SCD Erythroblasts

**Figure 1:**
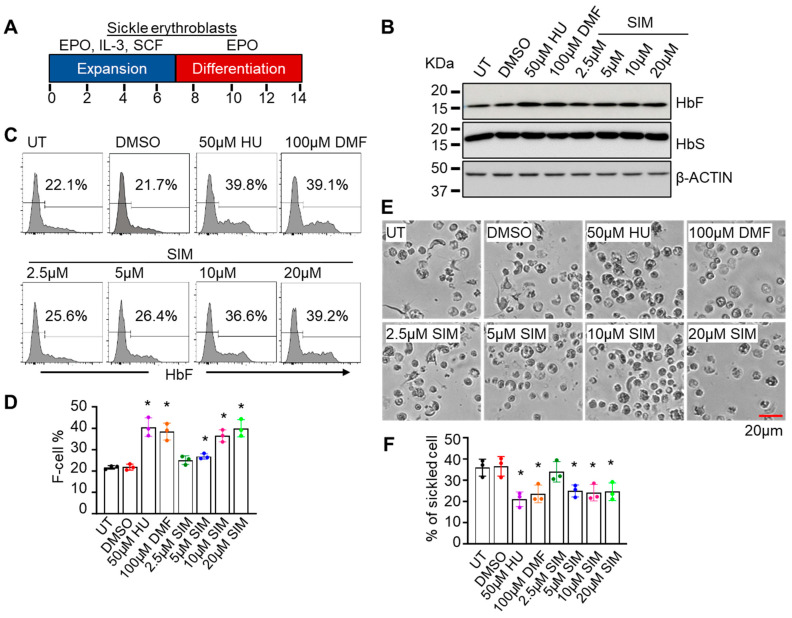
Simvastatin activates γ-globin expression to suppress sickling of SCD erythroblasts: (**A**) Two-phase culture of SCD patients’ peripheral blood CD34^+^ stem cells. (**B**) SCD erythroblasts at day 10 of culture were treated with 0.1% DMSO, 50 µM hydroxyurea (HU), 100 µM dimethyl fumarate (DMF), 2.5–20 µM simvastatin (SIM), or untreated control (UT) for 48 h and protein expression of fetal hemoglobin (HbF) and adult sickle hemoglobin (HbS) was quantified. (**C**,**D**) The percentage of HbF-positive cells (F-cells %) in SCD erythroblasts from the same culture was determined by flow cytometry (**C**) and quantified (**D**). (**E**) Cell morphology was analyzed to measure the number of sickled erythroblasts under hypoxia for the different treatment conditions and the percentage of sickled cells quantified (**F**). Data represent the mean ± SD of three independent biological replicates. *, *p* < 0.05.

Human sickle erythroid progenitors were cultured in a two-phase culture system (Figure 1A). On day 10 of culture, the cells were treated with the DMSO (0.05%) control, HU, DMF, or simvastatin at different concentrations for 48 h. Untreated cells (UT) were also set aside for control. We first determined cell cytotoxicity and growth under the different conditions. HU (50 μM), DMF (100 μM), and SIM (5–10 μM) showed no cytotoxicity but suppressed cell growth, compared to the UT and DMSO controls. Although SIM at lower concentrations did not affect cell growth or viability, SIM at 20 μM reduced both cell growth and viability in SCD erythroblasts (Supplementary Figure S1).

We next determined the effect of SIM on the production of fetal hemoglobin (HbF) and adult sickle hemoglobin (HbS) proteins. Although at the lowest concentration of 2.5 μM SIM slightly increased the HbF level, at higher concentrations of SIM (5–20 μM), HbF expression was significantly increased from 1.6- to 1.9-fold (Figure 1B); by contrast, HbS expression was not affected by SIM treatment. The percentage of HbF-expressing cells (F-cell%) was measured in parallel by flow cytometry. In agreement with the Western blot data, F-cell% was increased significantly with higher concentrations of SIM (5–20 μM) to 26–40%, an effect like that of HU and DMF treatments, compared to 22% F-cells with DMSO treatment (Figure 1C,D).

To further determine whether SIM could mediate an anti-sickling effect, SCD erythroblasts were incubated under hypoxia with different drug treatments. Similar to the effect of HU and DMF treatment, SIM produced anti-sickling effects, decreasing the number of sickled cells by 30–35% compared to DMSO and UT controls (Figure 1E,F). Together, these findings suggest that SIM induces HbF expression to modify the phenotype of sickle erythroblasts.

### 3.2. Simvastatin Increases the Expression of NRF2 and Antioxidant Proteins

**Figure 2:**
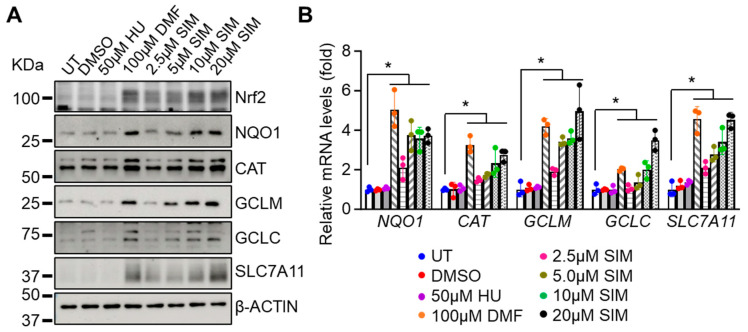
Simvastatin increases the expression of NRF2 and antioxidant proteins. Day 12 SCD erythroblasts after 48 h treatment with 0.1% DMSO, 50 µM HU, 100 µM DMF, 2.5–20 µM simvastatin (SIM), or untreated control (UT) were detected for the expression of antioxidant factors at the protein (**A**) and mRNA levels (**B**). Data represent the mean ± SD of three biological replicates. *, *p* < 0.05.

Previously, SIM was shown to upregulate NRF2 expression [38]. Recently, we demonstrated the significant role of NRF2 in regulating the sensitivity of SCD erythroid cells to ferroptosis in vitro and in mouse models of SCD. To extend these findings to sickle cells under oxidative stress, we first determined the NRF2 levels in SCD erythroblasts treated with different concentrations of SIM. A significant dose-dependent increase in NRF2 expression, like that produced by the known classical Nrf2 inducer DMF, was observed (Figure 2A). In addition, the expressions of classic downstream targets of Nrf2, such as the antioxidant proteins NAD(P)H dehydrogenase [quinone] 1 (NQO1), catalase (CAT), glutamate-cysteine ligase modifier subunit and catalytic subunit (GCLM and GCLC), and SLC7A11, were significantly increased at both the mRNA and protein levels by SIM in a dose-dependent manner (Figure 2). Notably, GCLC and GCLM are critical for the biosynthesis of oxidated GSH while SLC7A11 mediated the transport of cystine for GSH generation. Together, these results support the ability of SIM to induce the Nrf2 signaling pathway to activate transcription of γ-globin and antioxidant genes.

### 3.3. Simvastatin Enhances SCD Erythroblasts’ Antioxidative Capacity

**Figure 3:**
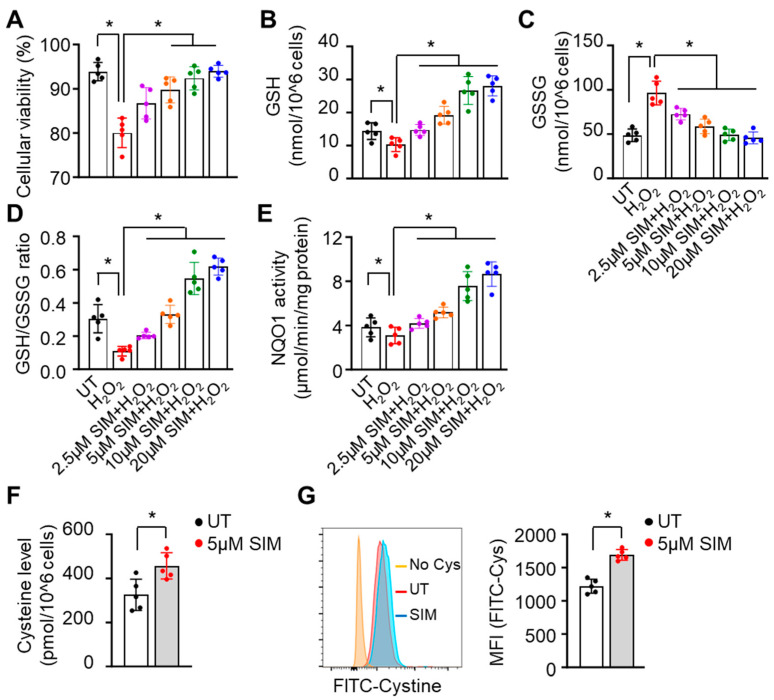
Simvastatin increased the antioxidant capacity of SCD erythroblasts. Incubation with H_2_O_2_ (40 µM) for 8 h was completed with day 12 SCD erythroblasts after 48 h treatment of 2.5–20 µM simvastatin (SIM), then cellular viability (**A**) and the cellular content of reduced (GSH), oxidative (GSSG) glutathione, and their ratio (**B**–**D**) was determined. The cellular NQO1 activity (**E**) and cystine contents (**F**) in the same cells cultured as described in panel A were measured with assay kits. (**G**) The cystine uptake efficacy of SCD erythroblasts was determined by a BioTracker Cystine-FITC Live Cell Dye followed by flow cytometry analysis. Data represent the mean ± SD of three biological replicates. *, *p* < 0.05.

We next determined whether the induction of Nrf2 signaling by SIM would affect the antioxidant capacity. SCD erythroblasts were treated with 40 μM of H_2_O_2_, which suppressed cell viability by about 20%; however, this suppression was rescued by simultaneous SIM treatment (Figure 3A). To investigate the effects of SIM on SCD erythroblast antioxidant capacity, we measured cellular reduced glutathione (GSH) and oxidative glutathione (GSSG) levels. Simvastatin significantly increased cellular GSH levels but did not affect GSSG, resulting in a higher ratio of GSH/GSSG (Figure 3B–D). Likewise, SIM increased NQO1 protein levels in a dose-dependent manner (Figure 3E).

Importantly, the availability of cysteine and its oxidative form cystine in the culture media contributes to the biosynthesis of GSH. A depletion of cystine in culture media leads to significantly reduced levels of GSH and increased oxidative stress [57]. Whether SIM affects the cystine levels required for GSH synthesis is not known. To determine an effect of SIM on cystine levels, we treated erythroblasts with 5 µM SIM, and found that cystine levels significantly increased by 40.4% (Figure 3F). To evaluate whether SIM affects cellular cystine uptake, we conducted a BioTracker Cystine-FITC assay using flow cytometry and found that SIM increased cystine uptake by 38.6% (Figure 3G). Together, these findings suggest that SIM can increase cystine uptake to promote an antioxidative effect.

### 3.4. Simvastatin Attenuates EZH2 Expression and Histone H3K27Me3 to Modify Chromatin Structure in Gene Regulation

**Figure 4:**
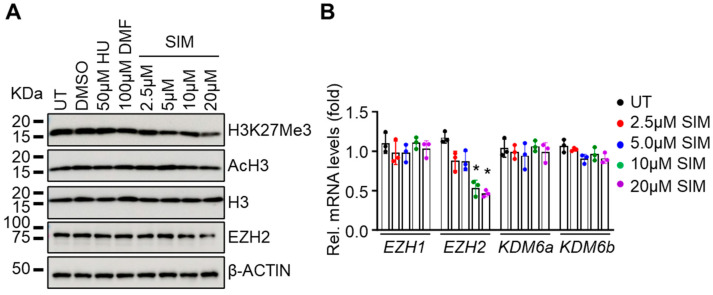
Simvastatin attenuates EZH2 expression and histone H3K27Me3 levels to modify chromatin structure and gene expression: (**A**) Shown are the effects of SIM on histone H3 methylation (H3K27Me3), acetylation (AcH3), and EZH2 protein expression. Total histone H3 and β-ACTIN were loading controls. (**B**) Quantitative RT-PCR analysis of the relative mRNA levels of H3K27Me3 modifiers *EZH1/2* and *KDM6a/b* in SCD erythroblasts treated with 2.5–20 µM simvastatin (SIM) or untreated control (UT). Data represent mean ± SD of three biological replicates. *, *p* < 0.05.

Previously, SIM was shown to downregulate histone lysine K27 trimethylation (H3K27Me3) through EZH2 and histone acetylation in a context-dependent manner [58,59]. To investigate whether SIM affects histone modifications in SCD erythroblasts, we determined global histone H3K27Me3 and histone H3 acetylation levels. Interestingly, though we did not detect an effect of SIM on histone acetylation, the level of the H3K27Me3 mark was significantly reduced (Figure 4A). Importantly, H3k27Me3 is regulated by the histone demethylases KDM6a/b and methyl transferases EZH1/2 [60]; however, we did not detect changes in KDM6a/b or EZH1 mRNA levels after SIM treatment (Figure 4B). By contrast, a significant reduction in EZH2 expression was detected (Figure 4A), suggesting that EZH2 is the downstream factor facilitating the ability of SIM to mediate histone H3K27Me3 modifications.

### 3.5. EZH2 Regulates NRF2 Expression to Modify ARE Motif Chromatin Structure on Target Genes

**Figure 5:**
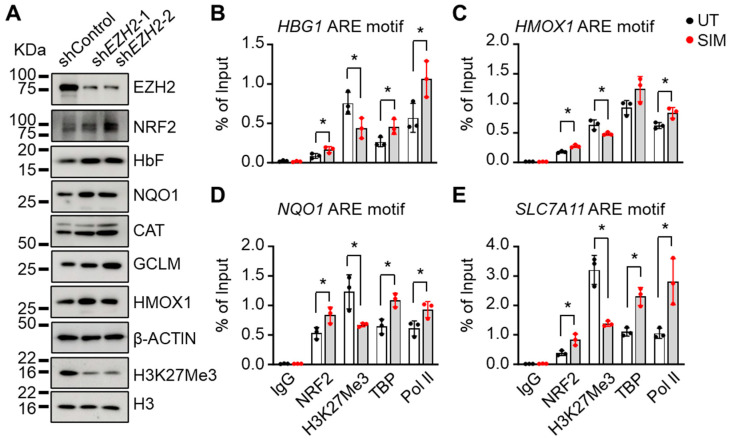
EZH2 regulates NRF2 expression to alter ARE motif chromatin structure in target genes: (**A**) The effects of sh*EZH2*-mediated gene silencing (two different constructs) on protein levels of NRF2, HbF, antioxidant factors, and H3K27Me3 were investigated. Histone H3 and β-ACTIN were loading controls. (**B**–**E**) ChIP assay determined the association of Nrf2, H3K27Me3, TATA box-binding protein (TBP), and RNA polymerase II (Pol II) to the *HBG1* (**B**), *HMOX1* (**C**), *NQO1* (**D**), and *SLC7A11* (**E**) gene loci for SCD erythroblasts treated or untreated (UT) with simvastatin (SIM, 5 µM). Primers spanning the ARE motifs of each gene were used. Data represent the mean ± SD of three biological replicates. *, *p* < 0.05.

Whether the global effects of SIM on H3K27Me3 levels will also affect NRF2 expression was subsequently determined. We knocked down *EZH2* gene expression by shRNA, which significantly decreased protein levels, along with reducing the levels of H3K27Me3 (Figure 5A), confirming the methyl transferase activity of EZH2. By contrast, the levels of NRF2, HbF, and the antioxidant proteins NQO1, CAT, GCLM, and HMOX1 were significantly increased in sh*EZH2* cells (Figure 5A).

Since NRF2 regulates antioxidant genes through ARE motif binding, we evaluated whether SIM affected antioxidant gene expression through EZH2-mediated Nrf2 regulation. By the ChIP assay, the chromatin structure in the ARE motifs of individual genes was determined. We observed that SIM treatment significantly increased NRF2 binding to the ARE motifs of different target genes, along with increased association of TBP and Pol II, but decreased H3K27Me3 (Figure 5B–E). Together, these findings suggest that EZH2 mediates NRF2 and downstream target gene expression through H3K27Me3 chromatin modifications.

### 3.6. In Vivo Treatment with Simvastatin Suppresses H3K27Me3 Modification in SCD Mice

**Figure 6:**
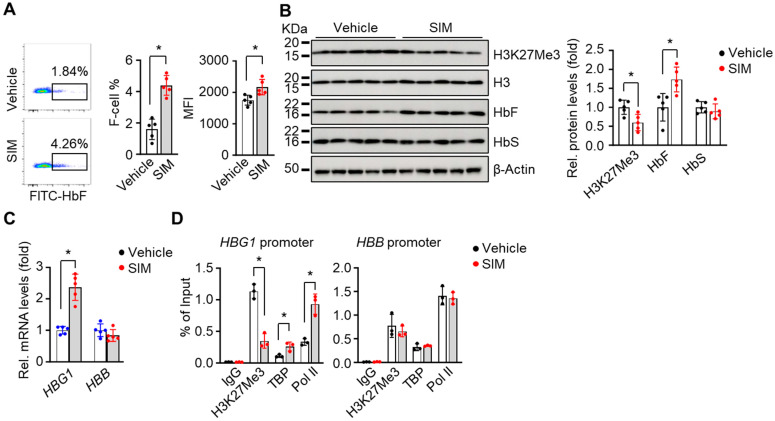
Simvastatin suppresses H3K27Me3 modifications to activate fetal γ-globin expression in preclinical SCD mice: (**A**) Peripheral blood samples of SCD mice after 4 weeks of simvastatin treatment were determined by the HbF-expressing cells (F-cells) by flow cytometry. The F-cell % and mean fluorescence intensity (MFI) of HbF-positive cells were quantified. (**B**,**C**) The spleen CD71^+^ cells from SIM-treated SCD mice were used to determine the expression of H3K27Me3, HbF, and HbS at the protein (**B**) and mRNA levels (**C**). (**D**) ChIP assay determined the association of H3K27Me3, TBP, and Pol II to the *HBG1* and *HBB* gene loci in spleen CD71^+^ cells from SIM-treated SCD mice. One-way ANOVA with Bonferroni’s multiple comparison tests was used for statistical analysis (n = 5). *, *p* < 0.05.

The in vitro findings support SIM-mediated γ-globin gene activation in SCD erythroblasts. To expand these results in vivo, we determined the ability of SIM to alter the SCD phenotype, using preclinical SCD mice treated with SIM (7.5 mg/kg) by daily intraperitoneal injects, 5 days a week for 4 weeks (a dose comparable to the 5 μM SIM concentration used in vitro). We first determined the hematological parameters of SCD mice under treatment conditions for which we did not observe significant changes between the SIM treatment and vehicle control, except for red blood cell counts (Supplementary Figure S2). However, F-cell% was remarkably increased in SCD mice treated with SIM, compared to vehicle controls (Figure 6A).

We next quantified histone and globin protein levels by Western blot of CD71+ erythroid cells isolated from spleen tissue. In vivo treatment with SIM decreased H3K27Me3 levels by 40.2% (Figure 6B), in agreement with the in vitro findings. Moreover, SIM induced HbF protein and its coding gene *HBG1*’s mRNA transcript levels but had no effects on the expression of HbS protein or its coding gene *HBB*’s mRNA transcript levels (Figure 6B,C), suggesting a gene-specific effect of SIM.

To further investigate the in vivo effect of SIM on chromatin structure, we conducted ChIP assays with SCD mouse spleen CD71+ cells. Of note, reduced H3K27Me3 levels were detected in the *HBG1* promoter after SIM treatment compared to the vehicle treatment control mice. In contrast, we did not observe differences for H3K27Me3 levels in the *HBB* gene promoter after SIM treatment (Figure 6D). Moreover, the association of TBP and Pol II in the promotion of the *HBG1* gene, but not that of the *HBB* gene, was increased (Figure 6D). These data support the ability of SIM to regulate H3K27Me3 modifications and chromatin structure as part of the mechanism of *HBG1* gene activation.

### 3.7. Simvastatin Protects against Organ Damage from SCD

**Figure 7:**
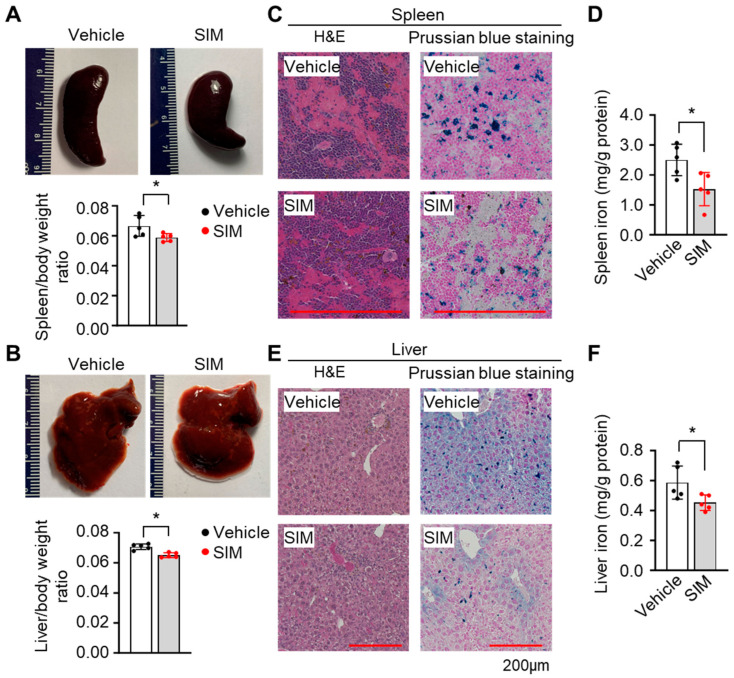
Simvastatin reduces organ damage and iron content in preclinical SCD mice: (**A**,**B**) Representative images of the spleen (**A**) and liver (**B**) of SCD mice treated with or without simvastatin (SIM, 7.5 mg/kg daily) for 4 weeks. Quantification of spleen and liver weights are shown in the graph. (**C**) Representative H&E and Prussian blue staining of SIM-treated SCD mouse spleens. (**D**) The iron content in spleen tissue was quantified. (**E**) Representative H&E and Prussian blue staining of SIM-treated SCD mouse livers. (**F**) The iron content in liver tissues was quantified. Scale bar, 50 μm. One-way ANOVA and Bonferroni’s multiple comparison test were used for statistical analysis (n = 5). *, *p* < 0.05.

The most common manifestation of SCD organ damage is splenomegaly, along with liver and kidney damage and increased iron deposition. Therefore, we examined these tissues to determine the effects of chronic SIM treatment on tissue phenotypes. We observed significantly reduced spleen and liver sizes when normalized by body weight (Figure 7A,B). Compared to the vehicle treatment, SIM treatment improved the histological structure of spleen tissue with a notable increase in white pulp staining (Figure 7C). Iron staining by Prussian blue showed that SIM reduced iron content by 39.1% (*p* < 0.05), suggesting less hemolysis in the spleen, compared to the vehicle controls (Figure 7D).

Likewise, there was structural liver damage and a substantial increase in iron for the vehicle-treated SCD mice according to H&E and Prussian blue staining, respectively (Figure 7E). By contrast, chronic SIM treatment significantly improved liver structure and reduced iron deposits by 22.9% (*p* < 0.05) compared to the vehicle controls (Figure 7E,F).

### 3.8. Simvastatin Reduces ROS Levels and Inflammatory Stresses in Preclinical SCD Mice

**Figure 8:**
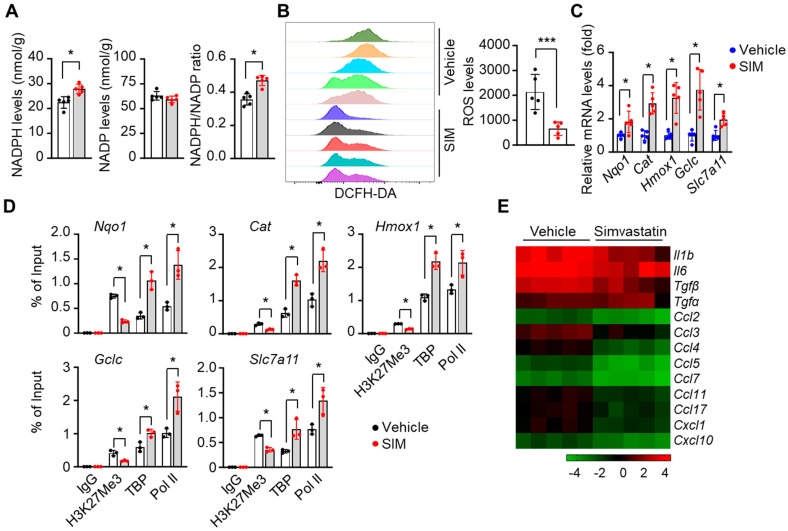
Simvastatin mitigates reactive oxygen species (ROS) and inflammatory stress in preclinical SCD mice. Cellular NADPH and NADP+ levels and their ratio (**A**), ROS levels by 2′-7′-dichlorodihydrofluorescein diacetate (DCFH-DA) staining (**B**), and relative expression of antioxidant genes (**C**) were determined in the spleen CD71^+^ cells of SCD mice. (**D**) ChIP assay determined the association of H3K27Me3, TBP, and Pol II to the antioxidant gene loci (*Nqo1*, *Cat*, *Hmox1*, *Gclc*, and *Slc7a11*) in spleen CD71^+^ cells of SIM-treated SCD mice. (**E**) Heatmap representation of pro-inflammatory factor gene transcripts in the peripheral blood of SCD mice between SIM and vehicle treatment control. One-way ANOVA with Bonferroni’s multiple comparison tests was used for statistical analysis (n = 5 mice). *, *p* < 0.05; ***, *p* < 0.001.

We next determined whether SIM affects ROS stress levels in spleen CD71+ erythroid cells. First, the quantification of cellular NADPH, NADP content, and their ratio showed that SIM increased NADPH levels by 24.3% and elevated the NADPH/NADP ratio by 32% (*p* < 0.05) (Figure 8A). Furthermore, the ROS levels determined by H2DCFDA flow cytometry showed that SIM reduced peripheral blood red cell ROS levels by >70% (*p* < 0.01) (Figure 8B). Notably, the expression of the Nrf2 targets Nqo1, Cat, Hmox1, Gclc, and Slc7a11 was manifestly increased after in vivo SIM treatment (Figure 8C). To further demonstrate that SIM upregulates antioxidant genes, we determined the chromatin structure in regulatory regions by the ChIP assay. Consistent with the in vitro findings, SIM reduced the association of repressive H3K27Me3 marks in the promoters of *Nqo1*, *Cat*, *Hmox1*, *Gclc*, and *Slc7a11* gene loci (Figure 8D).

It is well established that chronic hemolysis in SCD leads to increased free heme levels and inflammation [1,2], whereas Nrf2 activators such as DMF have been shown to alleviate inflammatory and oxidative stress [61]. Therefore, we determined the ability of SIM to suppress the expression of pro-inflammatory factors. Interestingly, a heatmap depiction shows that the mRNA transcript levels of inflammatory factors were decreased by 47.5–82.7% (*p* < 0.05), except *Tgfα*, which showed a 29.4% increase (*p* > 0.05) (Figure 8E). Nevertheless, RNA transcription profiles support an in vivo anti-inflammatory effect of chronic SIM treatment.

## 4. Discussion

Sickle cell disease is produced by a single nucleotide mutation in the adult β-globin gene leading to HbS synthesis and polymerization under hypoxic conditions, resulting in intravascular hemolysis, cell adhesion, vascular occlusion, and ischemia–reperfusion injury from elevated oxidative stress. In addition to multiple approaches for γ-globin activation [12,23], other strategies to treat SCD include targeting the downstream long-term effects by reducing oxidative stress, cellular adhesion mediated by P-selectin interactions, and increasing hemoglobin–oxygen affinity to directly block HbS polymerization. These four strategies have the potential to inform drug combinations to best treat the clinical complications and severity of disease in individuals with SCD. In this study, we have chosen to explore the possibility of repurposing the FDA-approved agent SIM, which stabilized Nrf2 protein levels and γ-globin activation by creating open chromatin domains and showed antioxidant and anti-inflammatory effects.

In the current study, we performed mechanistic studies which showed that SIM decreased global and gene-loci-specific H3K27me3 levels to affect heterochromatin for gene regulation in SCD erythroblasts (grown in culture) and SCD transgenic mice treated in vivo. This effect of SIM facilitated an activation of the γ-globin and antioxidant genes at the mRNA and protein levels to reduce red blood cell sickling and ROS stress, demonstrating its dual effect in modifying the phenotypic severity of SCD. Depending on the cellular environment, SIM can produce antioxidant effects by increasing the levels of reduced GSH such as in the liver [62], generate an pro-oxidative state in malignant conditions [63], or show no effect on oxidative stress regulation in healthy subjects [64]. Here, we demonstrated that SIM promotes the cellular uptake of cystine, the precursor for GSH biosynthesis, through Slc7a11 upregulation. In addition, both the catalytic (GCLC) and mediator (GCLM) subunits of the glutamate–cysteine ligase were upregulated by SIM to accelerate GSH biosynthesis and scavenging in ROS stress. Together, our findings and previous observations suggest a cellular-environment-dependent effect of SIM in regulating oxidative stress.

Previously, SIM was shown to inhibit histone deacetylase activity specifically targeting HDAC1/2 at a comparable capability to well-studied HDAC inhibitors such as Trichostatin A and suberoylanilide hydroxamic acid [47,65,66]. Simvastatin also mediates the induction of histone acetylation by activating the AMPK signaling pathway [59,67]. These functions of SIM through HDACs inhibition affect the expression of genes whose products participate in cell proliferation. In addition, SIM modifies the expression of key epigenetic proteins such as DNMT1 and influences DNA methylation to regulate gene transcription [68,69]. Our results in SCD erythroblasts showed that SIM did not change histone acetylation levels; however, SIM reduced the suppressive histone code H3K27Me3 through EZH2 activation, but not through EZH1 or the histone demethylases KDM6a/b. Although we did not explore the mechanism of SIM-mediated reduction in EZH2, a previous study demonstrated that SIM inhibits the function of METTL3 to downregulate EZH2 signals [70]. Those previous reports suggest cellular-environment-specific mechanisms of SIM in regulating histone modifications [71]. Of note, we previously showed that Nrf2 mediates metabolic reprogramming in erythroblasts to modify levels of the metabolite L-2-hydroxyglutarate, which is a competitor of α-ketoglutarate-dependent histone demethylases and thus regulates histone methylation [37]. Since we demonstrated that SIM enhances Nrf2 levels, our results raise the question of whether SIM could affect metabolic profiling, histone methylation, and gene expression through the Nrf2 signaling pathway in SCD erythroblasts. Future work will focus on answering this question.

From the ChIP assay analysis using primers spanning the gene-specific ARE motifs, our results support Nrf2 activation and binding to target ARE motifs to activate downstream target γ-globin and antioxidant genes through EZH2 suppression by SIM to produce an open chromatin structure and increase binding of the general transcription factors TBP and RNA Pol II. Previously, Nrf2 was shown to produce ARE-independent effects in regulating the expression of inflammatory factors, through binding to the proximity of the interleukin-6 and interleukin-1β genes to inhibit RNA Pol II recruitment and gene transcription in macrophages [72]. Though we are not able to exclude Nrf2 binding to regions devoid of ARE motifs to mediate γ-globin and antioxidant gene transcription, SIM reduced global and loci-specific H3K27Me3 levels, which led to chromatin structure changes at ARE motifs and other regions as supported by increased TBP and Pol II binding to promote gene transcription.

Related to the molecular function of EZH2, previous studies have shown the ability of EZH2 to act as a corepressor in cooperation with BCL11A to achieve γ-globin gene silencing [73]. Indeed, EZH2 is broadly distributed at high levels across the *HBB* locus in non-erythroid cells compared to erythroid cells, suggesting its general suppressive effect on globin gene transcription [74]. Moreover, the EZH2 inhibitor Tazemetostat can significantly increase fetal hemoglobin expression in healthy and β-thalassemic human primary erythroid cells [75]. Therefore, those observations and our finding of SIM-mediated EZH2/H3K27Me3 suppression to activate γ-globin transcription could expand the treatment options for SCD by inhibiting EZH2 expression.

In addition to activating HbF and antioxidant proteins, SIM suppressed pro-inflammatory signals in the SCD mouse model, which is in agreement with previous findings from human SCD clinical trials, where SIM administration reduced the markers of inflammation and the frequency of vaso-occlusive pain [76]. In a single-center pilot study of adolescents and adults with SCD, SIM treatment for up to 3 months led to a dramatic reduction in the rate of SCD-related pain and oral analgesic use; there was also improvement in soluble biomarkers of inflammation and an acceptable safety profile [77]. Inflammation and oxidative stress occur in chronic diseases and are closely correlated with each other, and are mutually influenced. Therefore, the failure of antioxidant drug trials might result from a failure to select appropriate agents that will target inflammation, and vice versa [78], whereas targeting both inflammation and oxidative stress simultaneously might generate synergistic effects. Importantly, oxidative and inflammatory stresses result from HbS polymerization and chronic hemolysis in SCD; therefore, SIM has the potential to produce a three-fold effect including HbF induction and inflammation and oxidative stress reduction to ameliorate the clinical severity of SCD.

In our study, simvastatin mediated HbF induction and Nrf2 activation, and suppressed inflammation effects in vivo in preclinical mouse SCD model when used at 5 µM or 7.5 mg/kg. Importantly, the current dose for simvastatin was between 20 and 40 mg/day for people with coronary heart disease to lower the low-density lipoprotein cholesterol levels [79]. Since simvastatin has been shown to cause muscle pain and calcium leak [80,81], the dose of simvastatin for the treatment of SCD would need to be optimized. Notably, we analyzed the effect of simvastatin on HbF induction in mouse spleen CD71+ cells under stress erythropoiesis, whereas in SCD patients, stress erythropoiesis occurred primarily in the bone marrow [82]. Therefore, additional studies to investigate the ability of simvastatin to induce HbF to ameliorate the clinical symptoms of SCD are warranted. In addition, there are notable differences in the liver iron content in the SCD mouse model and in patients with SCD, where mice exhibit iron overload whereas patients usually develop iron overload because of chronic blood transfusions.

Notably, a recent meta-analysis showed that statins can significantly decrease serum ferritin levels, which might be beneficial for the prevention and progression of atherosclerotic cardiovascular disease [83]. Indeed, iron overload causes inflammation and iron-dependent non-apoptotic cell death, i.e., ferroptosis. Individuals with SCD often suffer iron overload due to frequent blood transfusions, eventually requiring treatment with chelating agents to prevent liver and heart toxicity [84]. Our in vivo studies in SCD mice showed that SIM reduced the iron content in spleen and liver tissues. However, a link between chronic SIM use and iron deficiency was detected in humans [85]. Iron reduction by SIM is through the regulation of iron metabolism by HMOX1 expression [86]. We also observed an increase in HMOX1 expression in SCD mice treated chronically with SIM. Although at the SIM dose of 7.5 mg/kg/day we did not observe adverse effects in SCD mice treated up to 4 weeks, a lower dose of SIM might be more beneficial and should be evaluated.

## 5. Conclusions

In conclusion, our study presents compelling evidence for the ability of SIM to activate γ-globin gene transcription and decrease red cell sickling, oxidative stress, and inflammation. Importantly, our study indicates that through regulating histone H3K27Me3 levels and modifying EZH2 expression, SIM affects histone methylation status to alter chromatin structure and Nrf2 binding to regulate gene expression. This chromatin modification promotes the assembly of transcription complexes on the fetal γ-globin and antioxidant genes. Moreover, our findings support the ability of SIM to modulate iron and cystine/GSH levels while decreasing pro-inflammatory signals. Lastly, in vivo experiments demonstrated that SIM treatment improved phenotypic characteristics in preclinical SCD mice, suggesting potential therapeutic benefit.

## Data Availability

All relevant data are within the manuscript and the Supplementary Materials. Any additional information required to re-analyze the data reported in this paper is available from B.P. and X.Z. upon request.

## Supplementary Materials

The following supporting information can be downloaded at https://www.mdpi.com/article/10.3390/antiox13030337/s1: Table S1. List of antibodies. Table S2. List of DNA oligonucleotides. Figure S1. Effects of γ-globin inducers on cellular proliferation and viability of SCD erythroblasts. Figure S2. Peripheral blood hematological parameters of SCD mice treated with simvastatin.
