# Supplementary material for: Simvastatin-Mediated Nrf2 Activation Induces Fetal Hemoglobin and Antioxidant Enzyme Expression to Ameliorate the Phenotype of Sickle Cell Disease
Source: Antioxidants (Basel). 2024 Mar 11;13(3):337. doi: 10.3390/antiox13030337 (PMC10968127; doi:10.3390/antiox13030337)
Supplement: Supplementary file 1 [file antioxidants-13-00337-s001.zip › antioxidants-2874127-supplementary.pdf]

Supplemental Table S1, List of antibodies.

Mouse anti-β-ACTIN	Sigma-Aldrich	Cat# A5316
Rabbit anti-NRF2	Abcam	Cat# Ab62352
Rabbit anti-NRF2	Santa Cruz Biotechnology	Cat# SC-13032x
Rabbit anti-GCLC	Abcam	Cat# Ab41463
Rabbit anti-NQO1	Abcam	Cat# Ab80588
Rabbit anti-HMOX1	Abcam	Cat# Ab68477
Rabbit anti-NQO1	Abcam	Cat# Ab80588
Rabbit anti-Catalase	Santa Cruz Biotechnology	Cat# SC-50508
Rabbit anti-GCLC	Abcam	Cat# Ab41463
Rabbit anti-GCLM	Abclonal	Cat# A5314
Rabbit anti-SLC7A11	Cell Signaling Technology	Cat# 12691
Rabbit anti-EZH2	Sigma-Aldrich	Cat# 07-689
Mouse anti-adult hemoglobin (HbA)	Santa Cruz Biotechnology	Cat# SC-21757
Mouse anti-fetal hemoglobin (HbF)	Santa Cruz Biotechnology	Cat# SC-21756
Sheep IgG isotype FITC control antibody	MyBioSource	Cat# MBS524511
FITC conjugated mouse anti-HbF	BD biosciences	Cat# 552829
Donkey anti-Mouse IgG, secondary, HRP	Santa Cruz Biotechnology	Cat# SC-2096
Donkey anti-Rabbit IgG, secondary, HRP	Santa Cruz Biotechnology	Cat# SC-2077

Supplemental Table S2, List of DNA oligoes.

**cDNA analysis**	**Forward primer**	**Reverse primer**
β-*ACTB* (both human NM_001101 and mouse NM_007393)	GACGAGGCCCAGAGCAAGAGAGG	TCTCAAACATGATCTGGGTCATC
CAT (both human NM_001752and mouse NM_009804)	GGTTTGGCCTCACAAGGACTACC	CGGTAGGGACAGTTCACAGGTAT
GCLC (both human NM_001498 and mouse NM_010295)	GAGTATGGGAGTTACATGAT	TCTGGAAAGAAGAGGGACTT
NQO1 (both human NM_000903 and mouse NM_008706)	ATCCTGGAAGGATGGAAGAAAC	TCTAGCTTTGATCTGGTTGTCAG
GCLM (both human NM_002061 and mouse NM_008129)	AAGTGCCCGTCCACGCACAG	CTTCTAGTTGATGATGAAGA
SLC7A11 (both human NM_014331 and mouse NM_011990)	CTCCAGAACACGGGCAGCGT	CAAATGCCAGGGATATCACA
KDM6a (human NM_001291415)	TCACTTCAACCTCTTATTGGAAG	CTTTGAACATAAGCCCAAGTCGT
KDM6b (human NM_001080424)	TCCTCCTGCCACCAAAATACCTC	CCAGCCCATCAGGTAGGATCTTG

EZH1 (human NM_001991)	TGGTTCCCATCATGTATTCCTGG	GATCCAGGGATCATCTCTTCTTC
EZH2 (human NM_004456)	ATGGGCCAGACTGGGAAGAAAT C	GCGCAATGAGCTCACAGAAGTCA
HBG1 (human NM_000559)	GTGGATCCTGAGAACTTCA	GCTTGCAGAATAAAGCCTATCCTTG
HBB (human NM_000518)	GTGGATCCTGAGAACTTCA	TTAGGCAGAATCCAGATGCTCAAGG
Tgfb (mouse NM_011577)	GGTGCTCGCTTTGTACAACA	ACTGCTTCCCGAATGTCTGA
Tgfa (mouse NM_031199)	CATTGAGCCCACCCTTGTTC	ATGAGGAACAGGCAGCTTCT
Il1b (mouse NM_008361)	ACTCATTGTGGCTGTGGAGA	TGTTGTTCATCTCGGAGCCT
Il6 (mouse NM_031168)	CTGGGGATGTCTGTAGCTCA	CAGGTCTGTTGGGAGTGGTA
Ccl2 (mouse NM_011333)	AGGTGTCCCAAAGAAGCTGT	ACAGAAGTGCTTGAGGTGGT
Ccl3 (mouse NM_011337)	GATCTGCGCTGACTCCAAAG	GTCCCTCGATGTGGCTACTT
Ccl4 (mouse NM_013652)	CCTCTCTCTCCTCTTGCTCG	CTCTCCTGAAGTGGCTCCTC
Ccl5 (mouse NM_013653)	TGCCCACGTCAAGGAGTATT	AGATGCCCATTTTCCCAGGA
Ccl7 (mouse NM_013654)	TGAAGCCAGCTCTCTCACTC	TATAGCCTCCTCGACCCACT
Ccl11 (mouse NM_011330)	CACAAAGCACCTGGACCAAA	TCGTCCCATTGTGTTCCTCA
Ccl17 (mouse NM_011332)	GCTTCTGGGGACTTTTCTGC	TTGAAACCATGGACAGCAGC
Cxcl1 (mouse NM_008176)	AGTTCCAGCACTCCAGACTC	CTCGCGACCATTCTTGAGTG
Cxcl10 (mouse NM_021274)	ACATCAGCTGCTACTCCTCC	CTGAGCTAGGGAGGACAAGG
**ChIP analysis**	**Forward primer**	**Reverse primer**
HBG1 (human NM_000559)	CTGAAACGGTCCCTGGCTA	CTGTGAAATGACCCATGGCG
HBB (human NM_000518)	CACTTAGACCTCACCCTGTGGAG CCACACCCTAGGGTTGGC	CTTGTAACCTTGATACCAACCTG
HMOX1 ARE (human NM_002133)	CACGGTCCCGAGGTCTATT	AAGGGTGGAGGAGCTGCTAT
NQO1 ARE (human NM_000903)	ATTACCTGCCTTGAGGAGCA	CAGAGGCCTCAAAAATCTGG
SLC7A11 ARE (human NM_014331)	TGCATGAGGAAAATGATCCA	GCATGGAAAGGTCAGAACC
Nqo1 ARE (mouse NM_008706)	TTTCTAAGAGCAGAACGCAGCAC	AGACCTCCTGGGTACAAAATGGA
Cat ARE (mouse NM_009804)	GTAGGGAGAGACACGGTTTCCA	AGAAAAGACAAATCAAGAACCAG
Hmox1 ARE (mouse NM_010442)	AGTCACGGTCCCGAGGTCT	TCGAGTTGATCTTCTTACAGTG

Gclc ARE (mouse NM_010295)	CTCTTAACCGCTGAGCCATC	TATTGGGAGATGGGGAGAGA
Slc7a11 ARE (mouse NM_011990)	AAATTTCCGGCTTTGAGCTA	CTTGTCAGCACAGCGTGATT
**shRNA constructs**	**Forward primer**	**Reverse primer**
shControl	CCGGCCTAAGGTTAAGTCGCCCTCGCTCGAGCGAGGGCGACTTAACCTTAGGTTTTTG	AATTCAAAAACCTAAGGTTAAGTCGCCCTCGCTCGAGCGAGGGCGACTTAACCTTAGG
sh*EZH2* construct 1 (targeting human *EZH2* NM_004456)	CCGGTGACTTCTGTGAGCTCATTGCCTCGAGGCAATGAGCTCACAGAAGTCATTTTTG	AATTCAAAAATGACTTCTGTGAGCTCATTGCCTCGAGGCAATGAGCTCACAGAAGTCA
sh*EZH2* construct 2 (targeting human *EZH2* NM_004456)	CCGGGTTTGTTGGCGGAAGCGTGTACTCGAGTACACGCTTCCGCCAACAAACTTTTTG	AATTCAAAAAGTTTGTTGGCGGAAGCGTGTACTCGAGTACACGCTTCCGCCAACAAAC

Supplemental Figures

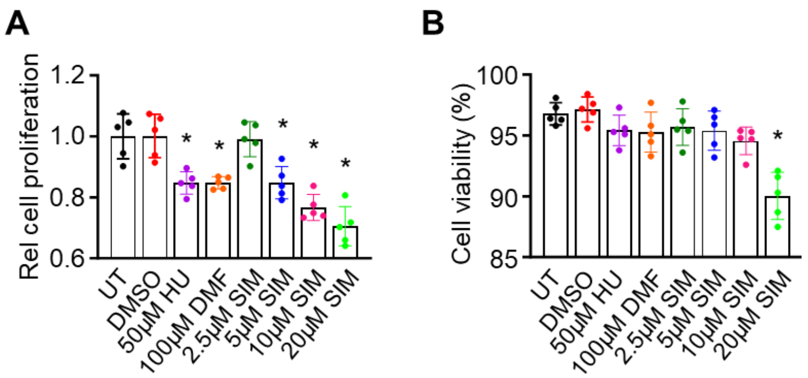

Figure S1, Effect of HbF inducers on cellular proliferation and viability of SCD erythroblasts. Cellular proliferation (A) and viability (B) of SCD erythroblasts at day 12 after 48 h of treatments with 0.1% DMSO, 50 µM hydroxyurea (HU), 100 µM dimethyl fumarate (DMF), 2.5-20 µM simvastatin (SIM), or untreated control (UT). Data represents mean ± SD of three biological replicates. *, $p < 0.05$.

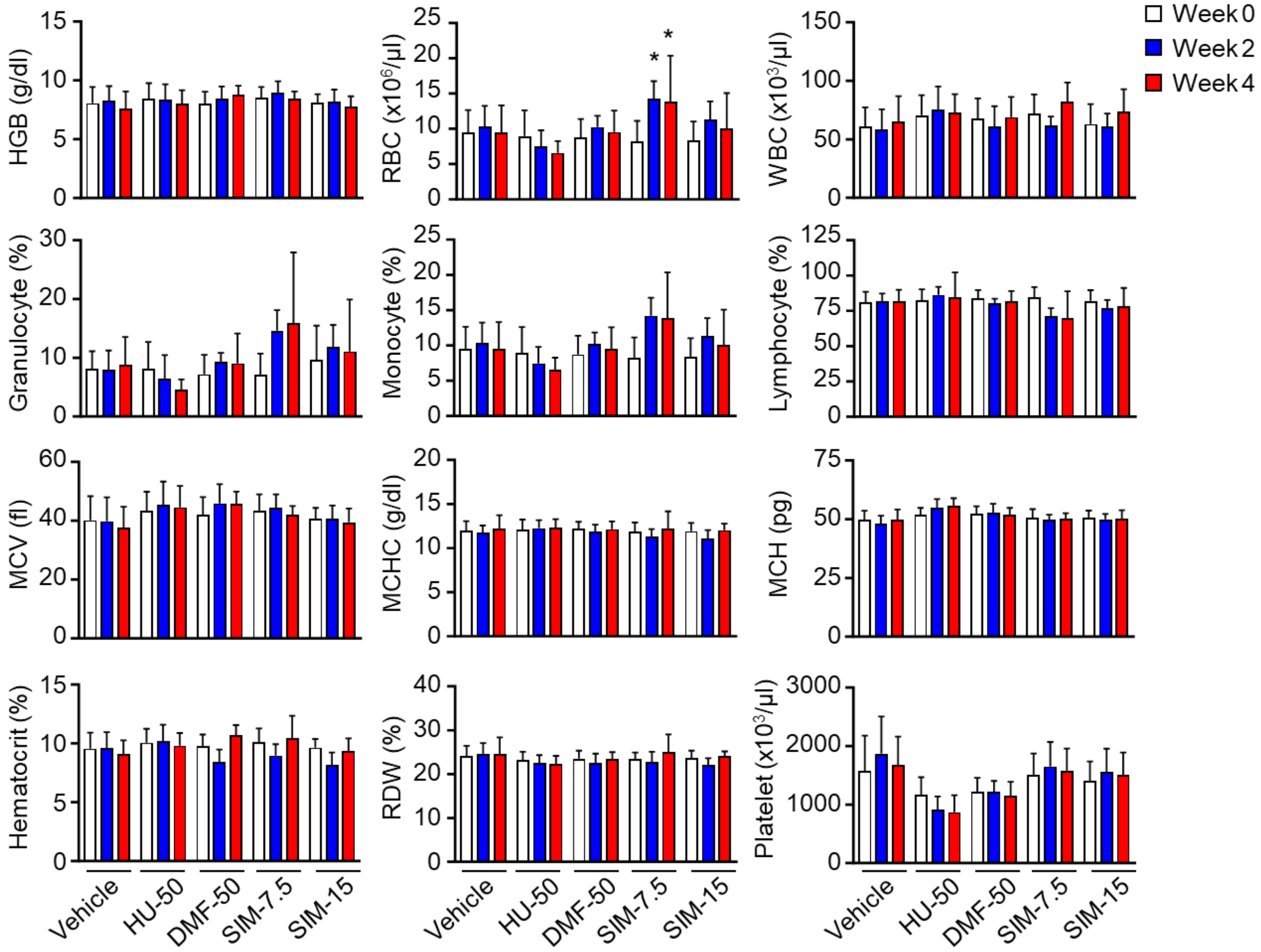

Figure S2, Hematological indices of SCD mice treated with simvastatin. Complete blood count and differentials for peripheral blood of the SCD mice, at week 0, week 2, and week 4 of treatment of simvastatin (SIM, 7.5-15 mg/kg), hydroxyurea (HU, 50 mg/kg) or dimethyl fumarate (DMF, 50 mg/kg) daily, 5 days a week. One-way ANOVA with Bonferroni's multiple comparison test was used for statistical analysis (n=5 mice). *, $p < 0.05$.
